# Emerging evidence that the mammalian sperm epigenome serves as a template for embryo development

**DOI:** 10.1038/s41467-023-37820-2

**Published:** 2023-04-14

**Authors:** Ariane Lismer, Sarah Kimmins

**Affiliations:** 1 Department of Pharmacology and Therapeutics, Faculty of Medicine, McGill University, Montreal, QC H3G 1Y6 Canada; 2 Department of Pathology and Cell Biology, Faculty of Medicine, University of Montreal Hospital Research Centre, Montreal, QC H2X 0A9 Canada

**Keywords:** Epigenetic memory, Epigenetics, Spermatogenesis

## Abstract

Although more studies are demonstrating that a father’s environment can influence child health and disease, the molecular mechanisms underlying non-genetic inheritance remain unclear. It was previously thought that sperm exclusively contributed its genome to the egg. More recently, association studies have shown that various environmental exposures including poor diet, toxicants, and stress, perturbed epigenetic marks in sperm at important reproductive and developmental loci that were associated with offspring phenotypes. The molecular and cellular routes that underlie how epigenetic marks are transmitted at fertilization, to resist epigenetic reprogramming in the embryo, and drive phenotypic changes are only now beginning to be unraveled. Here, we provide an overview of the state of the field of intergenerational paternal epigenetic inheritance in mammals and present new insights into the relationship between embryo development and the three pillars of epigenetic inheritance: chromatin, DNA methylation, and non-coding RNAs. We evaluate compelling evidence of sperm-mediated transmission and retention of paternal epigenetic marks in the embryo. Using landmark examples, we discuss how sperm-inherited regions may escape reprogramming to impact development via mechanisms that implicate transcription factors, chromatin organization, and transposable elements. Finally, we link paternally transmitted epigenetic marks to functional changes in the pre- and post-implantation embryo. Understanding how sperm-inherited epigenetic factors influence embryo development will permit a greater understanding related to the developmental origins of health and disease.

## Abstract (web summary)

Increasing evidence suggests parental environmental stressors can influence offspring health and disease outcomes. In this perspective, the authors provide an update of the paternal epigenetic inheritance field and propose links between paternally transmitted epigenetic influences and functional changes to the embryo.

## Introduction

How fathers transmit environmental information to their children to influence their development and long-term health remains unresolved. From both a disease prevention and evolutionary perspective, this is an intriguing area of research that crosses disciplines spanning toxicology, nutrition, reproduction, epigenomics and developmental biology. Interest in sperm has long focused on the process of spermatogenesis and its connections to infertility. Since the discovery of imprinted genes and their involvement in paternal effects in the next generation^1,2^, there has been a growing interest in epigenetic programming during spermatogenesis and its connection to offspring health.

Complex diseases (e.g. infertility, diabetes, metabolic disorders and cardiovascular disease), and developmental disorders (e.g. neurodevelopmental and birth defects) cannot be attributed to genetics alone. In humans, a myriad of environmental factors have been shown to impact an individual’s health (reviewed in^3^). The consequences of environmental exposures on health are widely influenced by gene x environment interactions, whereby certain genetic makeups or single-nucleotide polymorphisms (SNPs) predispose an individual to exhibit increased or decreased phenotypic sensitivities to distinct environmental stressors (^3^). Intriguingly, epidemiological studies and rodent models, show that paternal environmental stressors also affect disease risk in offspring^4–7^. Exposures to high fat diets, toxicants, or micronutrient deficiency can impact our health and that of future generations. Only now are we beginning to identify the molecular mechanisms that bridge these exposures in men to the development and health of their offspring^8–16^. One connection between environment and health is the epigenome. The epigenome refers to the biochemical content associated with DNA that dictates cell types, impacts gene expression and chromatin organization, and may be transmitted via the gametes to alter phenotypes across generations.

Over the past decade, epigenomics technologies have made remarkable progress, permitting in-depth and high-quality genome-wide analyses even at single-cell resolution^17–20^. These advancements have led to the direct implication of errors in the establishment and maintenance of the epigenome, in embryo development, neurodevelopmental disorders, diabetes and cancer^21–25^. How environment-induced changes to the sperm epigenome are transmitted at fertilization to persist in the embryo and drive phenotypic changes including birth defects and adult-onset of complex diseases that persist transgenerationally remains largely unknown. In human epidemiological studies, untangling the consequences of paternal environmental stressors on offspring health is challenging, due to our inability to fully rule out genetic factors in epigenomics datasets, and the difficulties in obtaining high-quality sperm and embryo cells.

Improving knowledge surrounding non-DNA sequence-based inheritance has the potential to lead to novel routes for the prevention and diagnosis of human diseases. Here, we provide an overview of the state of the field of intergenerational paternal epigenetic inheritance in mammals, and present new insights into the relationship between embryo development and the sperm epigenome. Of note, the phenomena of transgenerational epigenetic inheritance and reviews addressing the associations between environmental stressors and phenotypes across generations in mammals and other species has been covered extensively in^16,26–28^. Here, we first describe the intriguing functional logic between the retained epigenetic marks that make up the sperm epigenome and embryo development. Next, we delineate proven and potential mechanisms that serve as transmitters of paternal epigenetic information post-fertilization. We highlight the parallels between various environmental stressors, their impact on the sperm epigenome, and the resulting phenotype in the offspring. Finally, we link paternally transmitted epigenetic marks to functional changes in the pre- and post-implantation embryo. Understanding how sperm-inherited epigenetic factors influence embryo development will permit a greater understanding related to the developmental origins of health and disease.

## The final chromatin composition in the sperm of mice and men

### The histone-to-protamine exchange during spermatogenesis

In mammals, spermatogenesis is a stepwise process including proliferative mitosis and meiosis, followed by a massive remodeling of the chromatin that is unique to haploid phase of spermiogenesis^29–32^ (Box 1). Production of sperm requires tightly regulated transcription, chromosome recombination and epigenome programming. These steps depend on the regulation of histone modifications by the molecular machinery composed of readers and writers, and the histone-to-protamine transition. Also essential for spermatogenesis is the incorporation of histone variants including those that are testis-specific^29^ (H1T, H2A.X., H2A.Z., H2A-L1/2, HILS, H3T, TH2A, TH2B) (Box 1). Variants TH2B, H2A-L2 and H3T are postulated to induce nucleosome destabilization, facilitating chromatin remodelling^33–35^. Along with histone variants, transition proteins (TP1 and TP2) are incorporated into the chromatin as part of the remodeling process^36^. In mature sperm, 1% of histones are retained in mice and as much as 15% in men^37–40^. It was later determined that retained histones are conserved across species and found at important regulatory genes involved in housekeeping, spermatogenesis, and development^9,41–44^. More recently, chromatin enrichments at non-canonical loci have also been described^9,45–48^.

Historically, the principal function of sperm-specific protamines (P1 and P2) has been centered on their role in compaction of the paternal genome into the condensed sperm head. Flawed protamine incorporation or altered ratios is associated with impaired fertility^49^. Like histones, protamines undergo post-translational modifications including acetylation, methylation, and phosphorylation^50,51^. Suggesting a new role for protamines in embryo development, a rodent study showed that alanine substitution at lysine 49 on P1 leads to embryonic arrest^50^. Another study demonstrated that phosphorylation of sperm protamines by an oocyte splicing kinase SRPK1 was necessary to trigger protamine-to-histone exchange post-fertilization and initiate embryo development^52^. Whether modifications on protamines can be influenced by the paternal environment to impact embryo development remains a fruitful avenue for future investigation.

**Fig. 1:**
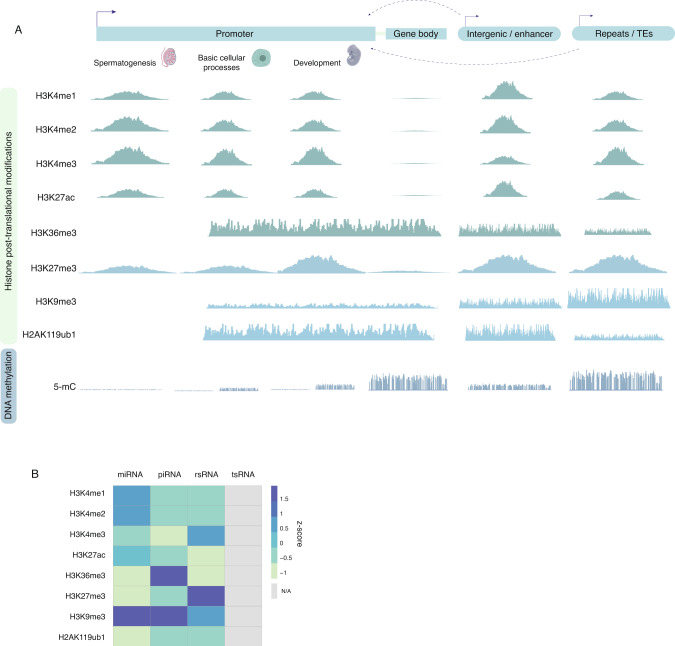
The final composition of the sperm epigenome prepares the embryo for developmental competency. **A** *A schematic showing the global epigenetic composition of sperm relative to functional loci of interest from the perspective of spermatogenesis and embryo development, based on whole-genome sequencing experiments*^21,31,37,42–46,48,54,63,114,169^*. For details on the dynamic nucleoprotein composition through spermatogenesis refer to* Box 1. Active and narrow marks in sperm including H3K4me1, H3K4me2, H3K4me3 and H3K27ac, are enriched at many promoters implicated in spermatogenesis, basic cellular processes, and development. Interestingly, these active marks are also enriched in intergenic space and putative enhancers. The enrichment of these marks relative to each other may influence function. Other marks such as the active H3K36me3, and the repressive H3K9me3 and H2AK119ub1, broadly cover certain gene bodies, promoters, and intergenic space, including transposable elements. Finally, although over 70% of the sperm epigenome is in a methylated state, DNA methylation remains mostly devoid at CpG dense promoters bearing nucleosomes. Panel created with BioRender.com. **B** *Non-coding RNAs coincide histone post translational modifications*. Z-scores between the relationship of non-coding RNAs and different histone post-translational modifications in sperm. Raw FASTQ files from the sperm PANDORA-seq small non-coding RNA dataset were downloaded^99^ (accession number GSE144666). PANDORA-seq small non-coding RNA datasets were aligned to the mm10 genome and annotated based on the SPORTS1.1 pipeline with one mismatch tolerance (github pipeline: https://github.com/junchaoshi/sports1.1). Classes of small non-coding RNAs are curated by SPORTS1.1. As an output, a FASTA file is obtained, and this file subsequently provides the specific small non-coding RNA sequences captured by the PANDORA-seq in sperm. The DNA sequence that coincided with different histone modification peaks in sperm was extracted, and whether the DNA sequence from the PANDORA-seq FASTA files overlapped to the DNA sequence from the histone modifications was determined.

Box 1 Spermatogenesis is characterized by dramatic chromatin and DNA methylation remodeling

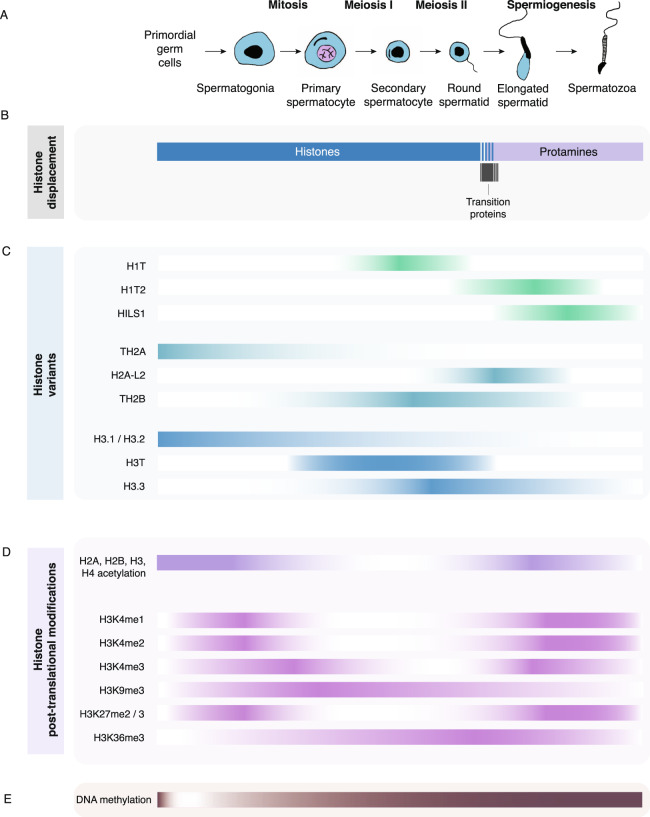

(A) Spermatogenesis is a highly complex cell differentiation process that encompasses rounds of mitosis and meiosis, accompanied by dramatic epigenome remodeling and genome compaction^171^. Spermatogonia type A stem cells are the most immature male germ cells and undergo asymmetric cell divisions to either maintain the stem cell pool, or give rise to spermatogonia type B which then proceed to generate primary spermatocytes. Primary spermatocytes undergo meiosis I, resulting in diploid secondary spermatocytes. After a second round of meiosis, haploid round spermatids develop. During spermiogenesis, round spermatids begin a condensation process, elongate their nucleus, acquire an acrosome, and obtain a flagellum. The resulting elongated spermatids mature to spermatozoa which are released into the lumen prior to their passage to the epididymis^172^.

(B) The first step of DNA packaging is initiated during spermatid development^173^ and involves a replacement of histones by transition proteins TP1 and TP2. Transition proteins localize to the nuclei of elongating and condensing spermatids^174^ and are required for normal chromatin condensation, protamine processing and sperm development. Protamines are small, arginine-enriched nuclear proteins that have most likely evolved from specialized histones^171^. Most mammals only express protamine 1 but mice and humans express two types of protamines, notably P1 and P2. Protamines are synthesized in elongating spermatids and their disulfide bonds are formed during the final stages of sperm maturation^175^. Both P1 and P2 are required for normal spermatid maturation and fertility^176^.

(C) H1 histones bind linker DNA to promote chromatin folding and compaction. The testes-specific H1T variant is found in pachytene spermatocytes and early haploid spermatids, making up over half of the linker histones during these stages. *H1t2* on the other hand, is expressed transiently during spermiogenesis and in elongating spermatids, localizing to chromatin of the apical pole, and is required for protamine placement^177,178^. HILS1 is a histone variant specific to spermiogenesis and most highly expressed in elongating and elongated spermatids^179^. HILS1 preferentially associates with LINE-1 elements in sperm^180^. Both H2A and H2B have testes-specific histone variants. TH2B is one of the earliest known histone variants in male germ cells to have been identified^181^. Synthesis of TH2B begins in early spermatocytes^182^ and its incorporation on chromatin follows a genome-wide removal of H2B. TH2B is required for the genome-wide replacement of histones by protamines^183^. H2A-L2 is another testes-specific histone variant expressed during late spermatogenesis at the time of histone replacement and its incorporation in spermatids in necessary for proper histone-to-protamine exchanges^184^. The histone variant H3.3 becomes preferentially incorporated into sperm chromatin relative to the H3.1 or H3.2 variants^37^. Finally, the testes specific H3T variant which becomes enriched in differentiating spermatogonia but is lost in spermatozoa, has been shown to regulate spermatogonial differentiation and ensure entry into meiosis by promoting an open chromatin structure^185^.

(D) Two global chromatin hyperacetylation events occur during spermatogenesis. H2A, H2B, and H4 become acetylated throughout spermatogonia to spermatocyte stages, and again in elongating spermatids (Fig. 1). However, histone acetylation returns to basal levels during meiosis of spermatocytes as well as in condensing spermatids^186^. Changes in histone deacetylases HDAC1 and HDAC6 levels were found to be fundamental for deacetylation maintenance or hyperacetylation initiation^186^. Because the acetylation of lysines within the N-terminal domains of histones decreases the histones’ positive charges and is thought to destabilize their affinity with DNA^187^, histone deacetylases may in part mediate histone eviction during spermatogenesis. Other main histone post-translational modifications involved in chromatin remodeling during spermatogenesis are represented. The data presented is based on immunohistochemistry and the intensity of color represents the strength of signal. Notably where no color is indicated does not mean the modification is not present, but rather may reflect limited chromatin accessibility for the antibodies used in Song et al.,^188^; Godmann et al.,^189^; Zuo et al.,^190^; Luense et al.,^191^; and Tatehana et al.,^192^. Many H3K4me2 regions in sperm are already present in spermatogonia at the same genomic loci, highlighting the early establishment of this mark during spermatogenesis. In fact, 44% of H3K4me2 regions in spermatogonia persist in sperm^44^.

(E) DNA methylation dynamics during spermatogenesis. Starting in spermatogonia, DNA methylation is acquired throughout spermatogenesis to attain an over 70% methylation state in sperm^67,69^.

### The sperm chromatin landscape is essential for embryogenesis

When interest in paternal epigenetic inheritance was burgeoning, it was unclear what histones were retained in sperm and whether specific modifications served a function beyond gene regulation during spermatogenesis. It was also entirely unknown whether histone modifications were sensitive to the paternal environment^29^. This initial lack of interest in sperm histones in the field of epigenetic inheritance was in part due to the knowledge that most sperm histones were evicted and replaced by protamines. However, the foundational findings that sperm histones mark genes implicated in embryo development suggested they may serve a function beyond gene regulation in spermatogenesis^42,43,53^. Histone H3K4me2 and H3K4me3 are overrepresented at sperm promoters and may serve different functional roles. H3K4me2 is found at promoters involved in spermatogenesis and cellular homeostasis^42^, whereas H3K4me3 co-localizes to promoters implicated in nuclear architecture, RNA metabolism, spermatogenesis, and embryo development^9,37,43^ (Fig. 1A). There is a high degree of conservation between mice and men for sperm H3K4me3 localization that corresponds to genes that are highly expressed in the early embryo^9,44,54^. Noteworthy is the over-representation of H3K4me3 in sperm from men at gene promoters harboring low complexity repeats and short interspersed nuclear elements (SINEs; Fig. 1A and Box 2), which are sequentially activated during early embryonic development^44,55^. In a landmark study we showed that disrupting the H3K4me2 landscape in sperm has dramatic consequences on the next generation^21^. Heterozygous transgenic male mice overexpressing the histone demethylase KDM1A in developing sperm gave rise to offspring with severe developmental defects. Surprisingly, KDM1A transgenic wildtype littermates sired by a transgenic male, but that did not bear the transgene, also sired abnormal offspring, indicating that the initial epigenetic disruption in the F_0_ generation was enough to propagate the phenotype for a subsequent 3 generations through the paternal germline. KDM1A functions by removing methyl groups from H3K4me1 and H3K4me2^56^, as well as H3K9me2 when associated with the androgen receptor in prostate cells^57^. In line with this, sperm from KDM1A transgenic males had reduced H3K4me2 enrichment at over 2000 transcriptional start sites at regions involved in development and morphogenesis^21^. Two-cell embryos from KDM1A transgenic males had differentially expressed genes that overlapped regions with reduced H3K4me2 in KDM1A transgenic sperm. Intriguingly, H3K4me2 was unaltered in the KDM1A transgenic wildtype littermate sperm, purporting that this histone mark was not responsible for the transgenerational phenotype observed in the offspring. More recently, H3K4me3 but not H3K27me3, was found to be altered in the sperm of both KDM1A transgenic and KDM1A transgenic wildtype littermate mice^54^. This finding demonstrates that in this model, altered H3K4me3 is propagated by a yet unidentified mechanisms down the paternal germline for multiple generations.

With improvements in sequencing technologies, the enrichment of sperm histones in mice and men has also now been detected in non-canonical intergenic and intronic space including at tissue-specific enhancers^9,44,45^ (Fig. 1A). Sperm H3K4me1 and H3K27ac overlap putative enhancers previously described in embryonic stem cells (ESCs)^45^, and H3K4me3 localizes to putative tissue-specific enhancers for limb, placenta, and testes^9^ (Fig. 1A). As H3K4me3 has been tightly associated with active promoters, the enrichment of sperm H3K4me3 in intronic or intergenic space could indicate its presence at alternative promoters, or unannotated promoters that become solicited during embryogenesis. This has been shown to be the case where H3K4me3 intergenic enrichment was identified at the intronic RLTR15 alternative promoter for the *Gab1* gene which is paternally expressed in the E7.5 placenta^58^. Genome-wide chromatin profiling in sperm reveals substantial overlap between H3K4me1, H3K4me3, and H3K27ac. Although H3K4me1 and H3K27ac typically mark active enhancers, they are also enriched at promoters in sperm^59^. In vitro studies show that the relative enrichment ratios between these marks are what distinguishes promoters from enhancers. It is therefore likely that balances between multiple histone modifications in sperm confers transcriptional and regulatory specificity in the embryo^60^. Therefore, disturbance associated with environmental exposures or infertility in these enrichment ratios could be a driver of paternal effects on embryonic development.

Although more enriched in intergenic space, the repressive H3K27me3 mark in sperm is also found at promoters^42,54^ (Fig. 1A). H3K27me3 is associated with developmental genes required for morphogenesis, ossification and organogenesis^42,54^. The co-occurrence of H3K27me3 and H3K4me3 is thought to poise developmental genes throughout early embryogenesis and ready them for expression later in post-implantation development^41^. In line with this, H3K27me3 is associated with gene repression in the pre-implantation embryo^37^. Examples of bivalently marked genes include the patterning *Hox* cluster genes, the limb development *Pax* genes, and the heart development *Hand* genes^43,54^. Studies have assessed whether the repressive H3K27me3 histone post-translational modification can also act as a mechanism for paternal epigenetic inheritance^61,62^. Male mice harboring two hypomorphic polycomb protein *Eed* alleles have compromised PRC2 function and a depletion of H3K27me3 enrichment in their fetal testis’ germ cells^61^. These mice are subfertile and sire litters with increased pregnancy losses. Regions with decreased H3K27me3 in their germ cells predominantly mapped to repetitive sequences, introns and intergenic space, and resulted in a derepression of long interspersed nuclear elements (LINEs). Both pre- and post-implantation embryo offspring from *Eed* hypomorphic males had abnormal gene expression at retrotransposed pseudogenes^61^ (Box 2). In another study, males with a germline-specific conditional knockout of the H3K27me3 and H3K27me2 demethylase KDM6A, sired offspring with reduced survivability and an increased susceptibility to tumor development^62^. The sperm of *Kdm6a* conditional knockout males showed redistribution of H3K27me3 at putative enhancers whereby H3K27me3 was decreased at regions of high H3K27me3 signal and increased at the flanking regions of lower signal. Regions with altered H3K27me3 in sperm were strongly associated with differentially methylated regions (DMRs), suggesting that the loss of sperm H3K27me3 sensitized these loci in the male germline. Strikingly, DMRs retained these methylation aberrancies in the somatic tissue of the next generation, highlighting a potential relay of information between sperm H3K27me3 changes and the more mitotically robust DNA methylation changes in offspring tissue^62^.

More recently, H3K9me3, H3K36me2/3, and H2AK119ub1 have been profiled genome-wide in developing germ cells and mature sperm^48,63,64^ (Fig. 1A). As opposed to the narrower H3K4me1/2/3, H3K27ac, and H3K27me3 marks that typically span 2 to 10 kb in sperm, H3K36me3 can mark entire gene bodies^42,54,63^. Interestingly, H3K9me3 and H2AK119ub1 are much broader and span up to 100 kb in sperm^46,48,63^. These repressive marks cover telomeric, centromeric, and peri-centromeric regions, and may be established during spermatogenesis. Their relation to protamine-mediated localization in the sperm genome and to sperm head compaction is unknown. H3K9me3 is associated with gene repression and marks numerous classes of transposable elements in sperm. Interestingly, H3K9me3-marked transposable elements lose H3K9me3 after fertilization on the paternal allele of the embryo, permitting their timely expression post-fertilization^46^. On the other hand, H3K36me2/3 is associated with active chromatin and functions in transcriptional fidelity, RNA splicing, and DNA repair^65^. In prospermatogonia, the lysine methyltransferase NSD1 deposits H3K36me2 in euchromatic regions which is critical for de novo DNA methylation establishment^64^. NSD1 germline deficiency leads to severe spermatogenic defects, and a failure to establish paternal imprints during spermatogenesis. Interestingly, NSD1-deficient females are fertile and exhibit no defects in oogenesis, highlighting the sex-specific consequences of improper H3K36me2 deposition and DNA methylation in mature gametes^64^. Finally, H2AK119ub1 stabilizes the polycomb subunits PRC1 and PRC2 in ESCs and instructs deposition of H3K27me3^66^. The effects of aberrant H2AK119ub1 during spermatogenesis and its implications in paternal epigenetic inheritance have yet to be uncovered.

Box 2 Classification of transposable elements and repeats Classification of (A) transposable element interspersed repeats and (B) tandem repeats, and the relative enrichment of main transposable element and repeat families in the mouse (mm10; m) or human genome (hg38; h). Classification tree was built based on the Dfam transposable element and repeat resource (https://www.dfam.org/classification/tree).
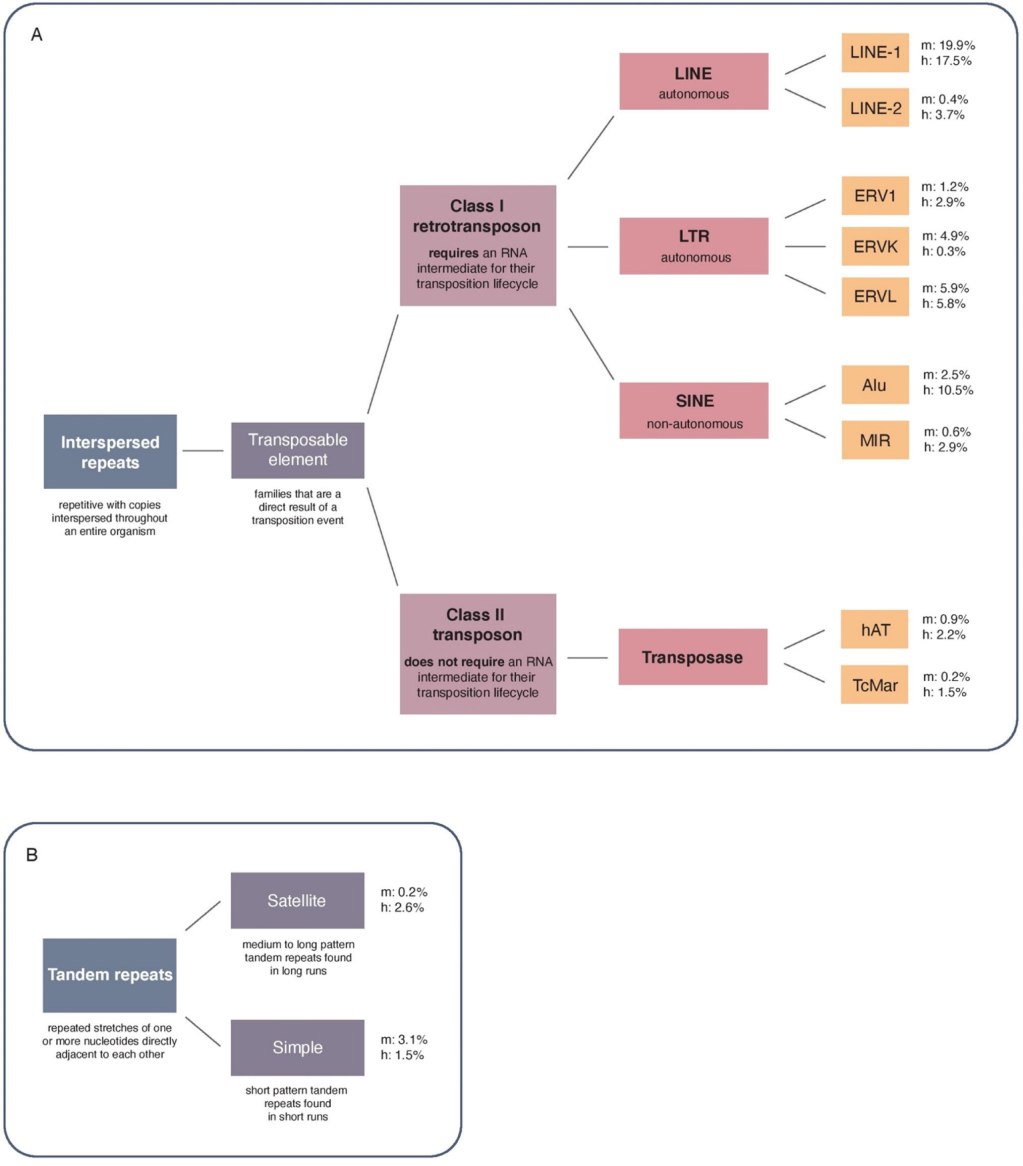

### DNA methylation is required during spermatogenesis and is linked to chromatin and non-coding RNAs

Although epigenetic modifications in sperm are frequently studied alone, there is functional interplay between DNA methylation, chromatin, and non-coding RNAs in the establishment and role of the sperm epigenome across generations. It is estimated that ~70% of DNA in mouse and human sperm is in a methylated state^67,68^ (Fig. 1A), and this widespread DNA methylation in sperm is critical for fertility and offspring viability^69–71^. In fact, de novo methylation in the mouse male germline by DNMT3A, DNMT3C, and the catalytically inactive co-factor DNMT3L, is essential for progression of spermatogenesis^72^. Indeed, disruption of DNMT3L leads to azoospermia^73^. In germ cells that lack DNMT3L, de novo DNA methylation at long terminal repeats (LTRs) and non-LTR retrotranposons (Box 2) fails to be established, leading to a reactivation of these TEs and meiotic arrest in spermatocytes^70^. Similarly, DNMT3C protects male germ cells by selectively methylating and suppressing young retrotransposons^74^. Lack of DNMT3C-mediated de novo DNA methylation also leads to failed meiosis in post-natal germ cells, aberrant chromatin landscape, and spermatogenic cell apoptosis. Finally, DNMT3A mutant males have impaired spermatogenesis, a lack of DNA methylation at paternally imprinted genes^75^, and spermatogonia stem cells are unable to commit to the spermatogenesis lineage^76^.

Promoters with high CpG content contain higher levels of nucleosomes than CpG-poor intergenic and intronic space, and are generally hypomethylated in sperm^37,43^ (Fig. 1A). Showing regulatory cooperativity between chromatin and DNA methylation machinery, peptide interaction assays have demonstrated that DNMT3L interacts with the extreme amino terminus of H3K4me to recruit a DNMT3A isoform for de novo DNA methylation. As H3K4me gains methyl groups, its interaction with DNMT3L become inhibited^77^. In vitro studies show that promoter CpG-rich regions with unmethylated DNA are preferentially bound by the H3K4me3 methyltransferases SET1A/B and MLL1/2. Intermediate DNA methylation levels reduce the binding of CpG-sensitive H3K4me3 methylases and promote H3K4me1-specific methylases, converting H3K4me3 to H3K4me1, to potentially influence enhancer priming^78,79^. This study suggests that DNA methylation balances H3K4me1 and H3K4me3 levels at promoters or enhancers to ultimately influence gene expression. Breaking the general misconception that histone H3K4me3 and DNA methylation are mutually exclusive in sperm, deep whole-genome bisulfite sequencing and H3K4me3 ChIP-seq of human sperm showed that over 24,000 H3K4me3 peaks overlapped regions marked by intermediate (between 20 and 80%) and high (over 80%) DNA methylation^44^. These overlapping regions occurred at developmental loci. Interestingly, the loci that bore H3K4me3 and high levels of DNA methylation lost their H3K4me3 and DNA methylation enrichments in the pre-implantation embryo and ESCs. Conversely, loci marked by high levels of H3K4me3 and low levels of DNA methylation in sperm retained H3K4me3 and became hypermethylated in the pre-implantation embryo^44^. These findings suggest that regions with low DNA methylation and high H3K4me3 levels may escape reprogramming post-fertilization. However mechanistic evidence in sperm and in the embryo confirming a co-functional role for their interaction is lacking.

An example of a co-functional relationship between the DNA methylation machinery interacting with RNA is exemplified by DNMT2 which is a tRNA methyltransferase implicated in paternal epigenetic inheritance^80^. Inactivation of *Dnmt2* was shown to prevent the transmissions of white tail phenotypes in *Kit* paramutants^81^, and of high-fat diet-induced metabolic disorders in male mice^82^. These models indicate the importance of tRNA methylation in the paternal transmission of phenotypes and suggest that methylation of these non-coding RNAs help stabilize them during the fertilization process.

### Sperm harbors a repertoire of non-coding RNAs that may be regulated by chromatin

There is extensive evidence that non-coding RNAs and chromatin are functionally connected in some cells and tissues but evidence of such interactions in sperm and embryos is lacking as studies of epigenetic inheritance have been singularly focused. Over the course of testicular spermatogenesis and post-testicular maturation in the epididymis, multiple waves of small non-coding RNAs are synthesized^83^. Indeed, piRNAs are enriched in fetal prospermatogonia^84^. During postnatal spermatogenesis, piRNAs are lowly abundant in pre-pachytene spermatocytes but become highly expressed again in pachytene spermatocytes and in post-meiotic round spermatids^84^. Approximatively 40–50% of prospermatogonia and prepachytene piRNAs originate from transposon-derived sequences, a higher enrichment than in the germ cells from later spermatogenic stages^85–88^. piRNAs interact with PIWI proteins to silence retrotransposons in the male germline through piwi-pathway-mediated DNA methylation. Mutations to components of the piwi pathway leads to spermatogenic catastrophe and arrest^89–92^. Upon sperm exit from the testis to its intro into the epididymis, there is then a dramatic switch between piwi-interacting RNAs (piRNAs) to transfer RNAs (tRNAs) and other non-coding RNA fragments^83^.

RNA-sequencing advances have revealed that sperm harbors an abundance of small RNAs that range from 29 to 34 nucleotides in size. A predominant amount of small RNA families in sperm match to the 5' half of tRNAs with cleavage sites that were preferentially located in an anticodon loop^93^. Based on classical RNA-sequencing approaches, these tRNA derived small RNAs (tsRNAs; 29 to 34 nucleotides in size) were estimated to constitute 65% of the sperm small-RNA repertoire. Micro-RNAs (15 to 25 base pairs in size) are the second most common class of small RNAs in sperm, making up 23% of total small non-coding RNAs^94^. A recently developed technique, Panoramic RNA Display by Overcoming RNA modification Aborted sequencing (PANDORA-seq), employs combinatorial enzymatic treatments to remove non-coding RNA modifications that block adapter ligation with reverse transcription during library preparation. This approach found a higher enrichment of sperm rRNA-derived RNAs (rsRNAs) than tsRNAs in comparison to the aforementioned studies which used conventional sequencing. Sperm is also enriched in long non-coding RNAs (>200 nucleotides in length) with the vast majority of unknown function^95,96^. An exception is *Tug1* which is required for male fertility^97^.

To date the relationship in sperm between non-coding RNAs, DNA methylation, and chromatin, is largely unexplored. This is due in part because current non-coding RNA aligner tools focus on identifying different classes of non-coding RNAs rather than mapping them to specific loci in the genome^98,99^. Determining the genomic origins of sperm non-coding RNAs will provide insight on their regulated expression, functionalities, and how they may be associated with sperm chromatin features and DNA methylation. To explore the potential relationship between non-coding RNA and specific chromatin modifications we used sperm PANDORA-seq and histone modification datasets and found that certain histone modifications coincide with specific classes of non-coding RNAs^99^ (Fig. 1C). Sperm micro-RNAs preferentially map to regions bearing the activating H3K4me1 and H3K4me2 marks whereas rsRNA overlap regions of bivalent H3K4me3 and H3K27me3 chromatin. Intriguingly, sperm tsRNAs do not coincide with any histone modifications peaks, potentially due to the smaller fraction of tsRNAs identified in sperm heads (representing <20% of non-coding RNA classes according to PANDORA-seq compared to 65% in classical RNA-seq approaches). The overlap between non-coding RNAs and chromatin features suggests a regulation of non-coding RNAs by histone modifications. In line with this possibility, transcription of the long non-coding RNA *Maenli* affects the deposition of H3K4me3 at *En1*^100^. Deletion of either *Maenli or En1* leads to limb defects in mice and humans due to a loss of expression of the *En1* gene, which is located in the same topological associated domain as *Maenli*.

The interaction of micro-RNAs with chromatin components is well documented; micro-RNAs can interact with various epigenetic remodelers to reshape the chromatin landscape in the context of cancer and development. In human prostate cancer, loss of micro-RNA-101 leads to the overexpression of the histone methyltransferase EZH2, a global decrease in H3K27me3, and cancer progression due to loss of repressive marks at tumor suppressive genes^101^. Micro-RNA-9 and 124 are critical to suppress the chromatin remodeler BAF53A, a subunit of the neural-progenitor-specific BAF complex. Mutation in these non-coding RNA sequences leads to lack of dendritic outgrowth and reduced proliferation in mouse embryo neurons^102^. Whether micro-RNAs similarly reorganize the chromatin landscape during spermatogenesis and in pre-implantation embryogenesis is unknown.

## Epigenetic mechanisms of transmission from sperm to the pre-implantation embryo

Although there is a clear parallelism between environmentally sensitive regions of the sperm epigenome and phenotype in the offspring (Box 3), a requirement for demonstrating mechanistic underpinnings is to show their capacity to be transmitted and escape epigenomic reprogramming to impact embryonic gene expression. Identifying such mechanisms has been elusive. However, with advances in low-input chromatin and DNA methylation profiling, significant progress towards the identification of sperm-inherited marks in the pre-implantation embryo has been made.

Box 3 The sperm epigenome is sensitive to environmental stress at functional regions that correlates with phenotypes in the offspring

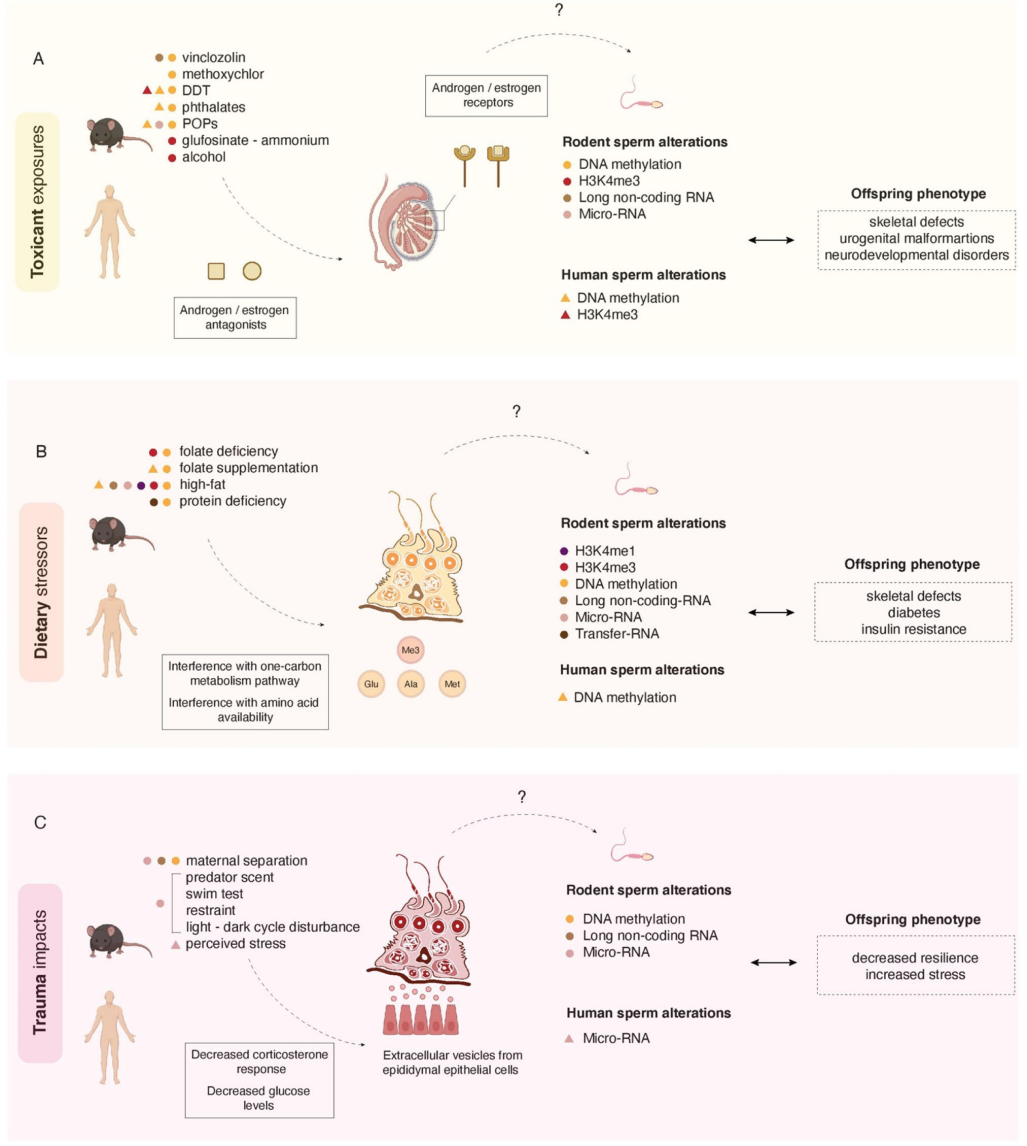

Environmental exposures in rodents and men are associated to distinct phenotypes in their offspring, suggesting that the environmentally sensitive regions in sperm carry direct consequences in the development of the next generation. A major limitation of studies to date on environmental exposures and epigenetic inheritance is the difficulty in progressing to mechanisms of transmission that go beyond correlation. Figure created with BioRender.com.

(A) *Paternal toxicant exposures and epigenetic inheritance*. A landmark study in the field of epigenetic inheritance demonstrated that male rats exposed to the pesticides vinclozolin or methoxychlor beginning in utero^193^ exhibited subfertility which was propagated transgenerationally. This transgenerational phenotype was attributed to altered DNA methylation in testicular cells^193^. Since then, other toxicants have been shown to induce epigenetic changes in the sperm of exposed male rodents and men^114,194–200^. The aforementioned toxicants are classified as endocrine disruptors (EDC) and perturb cellular homeostasis by behaving as androgen or estrogen antagonists^201^. It is worth noting that a direct mechanism to epigenetic changes in sperm may be meditated by EDCs targeting androgen and estrogen receptors that are bound to open chromatin loci in sperm^147^. A second potential mechanism whereby EDC induce methylation changes to histones and DNA methylation in sperm could be via androgen regulation of enzymes that participate in the one carbon metabolism such as glycine *N*-methyltransferase, cystathionine β-synthase, and ornithine decarboxylase. Such action would alter methyl donor availability and this may in part explain how endocrine disruptors can impact methylation of DNA and histones in sperm^202^. Mounting evidence indicates that steroid receptors also contribute to epigenetic cellular changes by interacting with chromatin remodelers. For example, the histone demethylase KDM1A can associate with the androgen receptor to target H3K9me2 and modify certain components of the chromatin landscape^203^. In the context of cancer, androgen and estrogen receptors have been shown to bind to different chromatin remodelers and promote cancer progression. For example, the histone demethylase JMJD2B (KDM4B) interacts with the estrogen receptor and demethylates H3K9me3 to permit the expression of estrogen-receptor sensitive genes, leading to breast cancer proliferation^204^.

*(B) Paternal diet and epigenetic inheritance*. Interest in the role of diet in the inheritance of complex disease was in part initiated by an epidemiological study of the effects of ancestral food availability on health and disease across generations. Using regional records from a population in Överkalix Sweden on food availability during male puberty, researchers linked an overabundance of food with a shortened lifespan and increased risk of disease in descendants^4^. This opened the possibility that complex disease may be influenced by paternal diet such as folate deficiency, high-fat diet, and low protein diet, and be transmitted across generations^8–11,83,94,205^. Folate availability impacts one-carbon metabolism, which in turn can influence DNA methylation and chromatin remodeling. Altering folate levels in male mice as shown in folate deficiency or folate supplementation models (B9) impacts H3K4me3 and DNA methylation in sperm. A high-fat diet and protein deficient diet may also impact the folate cycle and downstream methyl donor availabilities.

(C) *Trauma as a trigger for epigenetic alterations in sperm and heritable behavior*. A controversial and complex biological question is whether a parent’s emotional experiences can be transmitted to their children to impact behavioral disease risk. Male mice exposed to a variety of traumatic stressors including cage disruptions, predator scent, restraint, cold water swim, maternal separation, have decreased corticosterone response following stress^140,141^. Exposures to stress via early maternal separation has also been shown to decrease glucose levels in the blood^51,95,96^. A working hypothesis on how sperm senses changes in corticosterone is through the transmission of soma-to-germline transmission of microvesicles. Due to current technical limitations for isolating pure epididymal extracellular vesicles, a group developed an in vitro culture system which aimed to understand how these vesicles sense stress hormone changes and relay the information to sperm. Caput-derived sperm were isolated and incubated with either non-treated or corticosterone-treated mouse caput epididymal epithelial cells. Following intracytoplasmic sperm injection, offspring from sperm incubated with the corticosterone-treated cells had gene expression alterations in their brains and placentas in pathways involved in signaling and neurotransmitter transport^141^. Another possible molecular mechanism whereby stress could trigger epigenetic alterations to the sperm epigenome is increased levels of cortisol in the semen^206^ or via glucocorticoid receptors in the somatic and germ cells of the testis^207^.

### Evidence for transmission and persistence of sperm chromatin signatures to the embryo

Altering the histone modifiers genetically or via environmental exposures during spermatogenesis, in combination with modifying histone residues in the paternal pronucleus of the zygote, has provided strong evidence that sperm-inherited histones play an important role in development^8,9,21,61,62,103,104^. A clear indicator that sperm histones are transmitted at fertilization and retained in the embryo is the localization of sperm-enriched histone variant H3.3 to the paternal pronucleus of the zygote^103,104^. Overexpression of mutant H3.3 K27 provoked a derepression of zygotic paternal heterochromatin, resulting in a global decrease of H3K27me1 and an accumulation of major satellite transcripts in the embryo^103^. Likewise, loss of H3K4me1 and H3K4me3 in the paternal pronucleus via overexpression of mutant H3.3 K4, induced a decrease in the minor zygotic activation wave and caused developmental retardation during pre-implantation embryogenesis^104^. Although these studies do not demonstrate a direct transmission of sperm chromatin to the embryo, studies involving allele-specific breeding schemes have; a greater retention of H3.3 occurred on the paternal allele compared to the maternal allele of the pre-implantation embryo^105^. During oogenesis, non-canonical H3.3 patterns gradually form and by the 2-cell stage, oocyte-inherited non-canonical H3.3 patterns are lost to instead mirror the canonical H3.3 patterns inherited from sperm on the paternal allele^105^.

Whether sperm histones undergo complete reprogramming during embryogenesis has until recently been unclear. One study postulated that sperm-inherited H3K4me3 was erased on the paternal allele of the pronuclear stage 5 pre-implantation embryo and re-established in the 2-cell embryo at the same genomic loci than in sperm^106^. Re-analysis of this dataset demonstrated that the paternal allele of the PN5 zygote maintains 37% of regions with H3K4me3 from sperm^9,54,107^. This finding highlights that sperm chromatin is not completely reprogrammed after fertilization and entails that alterations in sperm histone modifications may be transmitted to the pre-implantation embryo to alter its gene expression. Other marks such as H3K27me3, H3K36me3, H3K9me3, and H2AK119ub1, appear to retain a negligible number of sperm-inherited peaks on the paternal allele from sperm to the PN5 zygote^46,48,63,108^. It is worth considering that this may be an artificially reduced amount from the lack of reads mapping to the paternal allele due to limitations of low-input sequencing^106^. Consequently, it remains challenging to conclude whether a sperm-inherited histone modification has undergone epigenetic reprogramming in the pre-implantation embryo, or if the absence of a chromatin enrichment is due to the limitations of ultra-low-input ChIP-seq and bioinformatics maternal / paternal SNP assignment techniques. Nonetheless, paternally inherited H3K9me3 carries clear functional relevance in the embryo. Paternally and maternally H3K9me3 imprinted regions were cataloged in the pre-implantation embryo at loci with high DNA methylation using allele-specific analyses^109^. To demonstrate their parent-of-origin functionalities, the authors knocked out certain H3K9me3 imprinted regions in male or female mice and assessed the effects on their offspring. Knock-out of H3K9me3 on paternally imprinted mCHM_177, impacted embryo development when passed through the male germline but not through the female germline^109^.

**Fig. 2:**
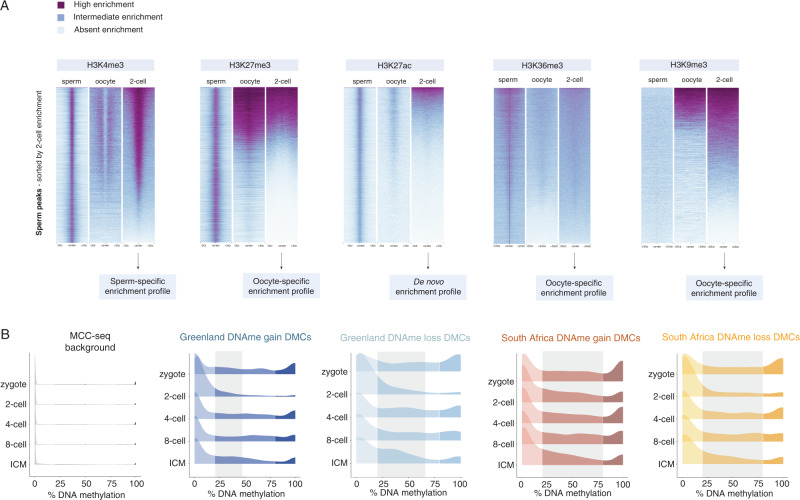
Chromatin marks in sperm and their relationship to the oocyte and 2-cell embryo. **A** *Heatmaps indicating H3K4me3, H3K27me3, H3K27ac, H3K36me3, or H3K9me3 regions in sperm and these marks’ enrichment in the oocyte and in 2-cell embryos*. Color of the signal corresponds to relative RPKM counts. Sperm H3K4me3 and H3K27me3 datasets were generated from Lismer et al.^54^; oocyte H3K4me3 dataset was retrieved from Liu et al.^110^; 2-cell H3K4me3 dataset was retrieved from Liu et al.^110^. Sperm H3K27ac dataset was generated in house; oocyte and 2-cell H3K27ac datasets were retrieved from Dahl et al.^111^. Sperm, oocyte, and 2-cell H3K36me3 datasets were retrieved from Xu et al., 2018^63^. Sperm, oocyte, and 2-cell H3K9me3 datasets were retrieved from Wang et al.^46^. Raw FASTQ files were aligned as indicated in Lismer et al.^9^. Regions with histone post-translational histone enrichments in sperm were identified using the *csaw* Bioconductor package^170^. Heatmaps were generated using the DeepTools software. **B** *DNA methylation (DNAme) is altered in sperm from DDT-exposed Greenlandic Inuit and South African Vhavenda men at regions that predicted to persist in the developing embryo*^114^. Ridge plots corresponding to the density of low, dynamic, or high CpG DNAme levels (*x*-axis) for different stages of pre-implantation embryo development (y-axis) at CpGs overlapping MCC-seq background (gray), Greenlandic sperm DNAme gain DMCs (dark blue), Greenlandic sperm DNAme loss DMCs (light blue), South African sperm DNAme gain DMCs (red), and South African sperm DNAme loss DMCs (yellow). Gray shaded boxes correspond to DMCs that retain dynamic CpG levels throughout pre-implantation embryogenesis. See Lismer et al., 2022 for full experimental details.

To further explore the putative contributions of sperm chromatin to the embryo, we used existing data sets to call histone modification peaks in sperm and profiled the histone enrichments at the same loci in 2-cell embryos and oocytes (Fig. 2A). This identified the potential for preferential sperm or oocyte mediated epigenetic inheritance^46,63,110^. For example, H3K27me3, H3K36me3 and H3K9me3 patterns in 2-cell embryos resembled the oocyte broad chromatin domains rather than the enrichments found in sperm suggesting a greater oocyte transmission^46,63,110^. In comparison, the H3K27ac profile appears to be established de novo in the 2-cell embryo and carries no similarities to either male or female gametes^111^. Finally, H3K4me3 peaks from sperm significantly overlap H3K4me3 peaks in the 2-cell embryo, which appear to be mutually exclusive from the H3K4me3 domains found in the oocyte suggesting paternal transmission^110^ (Fig. 2A).

While the function of the aforementioned histone modifications when maternally or paternally transmitted has not been fully explored, there is evidence of a preferential paternal transmission of H3K4me3 from environmental exposures studies (Box 3). Specifically, evidence that sperm H3K4me3 serves as a metabolic sensor is demonstrated in mouse models where certain regions in the sperm of folate deficient or sperm from obese sires, showed H3K4me3 alterations at developmental and metabolic genes^8,9^. In the folate deficient model, 25% of the abnormal H3K4me3 at promoters in sperm retained their aberrancies in 8-cell embryos and were associated with a corresponding deregulated gene expression^8^ (Box 3). Likewise, the herbicide glufosinate-ammonium altered sperm H3K4me3 and H3K27ac at immune and nervous system genes, and the deregulated genes in the 4-cell embryo overlapped H3K4me3-altered regions in the sires’ sperm^112^. Still further evidence that sperm H3K4me3 serves as an environmental sensor that influences embryo development, was shown in a paternal alcohol exposure mouse model. Alcohol exposure increased H3K4me3 at neurogenesis and craniofacial promoters in sperm and led to correlated increases in CTCF in next generation placentae at the same genomic loci^113^.

While these studies are based on animal models, it has recently become clear that H3K4me3 is also sensitive to environmental perturbations in human sperm. In sperm from DDT-exposed Indigenous Greenlandic and South African men, H3K4me3 enrichment was altered in a dose-like response at young transposable elements, neurodevelopmental genes, and putative embryonic enhancers^114^. Interestingly, children born in DDT-exposed populations exhibit higher rates of attention deficit and hyperactivity disorders, autism and neurodevelopmental delays^115–118^. Taken together, these studies emphasize that H3K4me3 is a bone-fide mediator of paternal epigenetic inheritance that can drive changes in gene expression in embryos as a consequence of environmental exposures. Nonetheless, as ChIP-seq is an antibody-based technique, careful considerations should be adopted for quantification of sperm and pre-implantation embryo datasets. Indeed, effectuating antibody titrations to optimize signal to noise ratios, and use of proper batch designs, exogenous spike-in calibrations, high sequencing depth, and bioinformatics normalization tools, can all aid biologically-relevant ChIP-seq readouts.

### Evidence for transmission and persistence of sperm DNA methylation in the embryo

During mammalian embryogenesis, two waves of DNA demethylation take place^72^. These genome-wide reprogramming events are thought to provide plasticity to the developing embryo and allow germ cells to re-acquire DNA methylation in a sex-specific manner. Initial regions of sperm DNA methylation shown to be transmitted to the embryo and required for proper development include imprinted genes, and certain classes of transposable elements. Development of reduced-representation bisulfite sequencing (RRBS) and more recently whole genome bisulfite sequencing (WGBS) has provided novel insights regarding DNA methylation dynamics during spermatogenesis and embryogenesis, at single-nucleotide resolution^119–122^. Consequently, it has been shown that in addition to imprinted genes and transposable elements, there are other functional regions of the paternal methylome that may resist reprogramming in mice and men^119–122^ (Fig. 2B).

**Fig. 3:**
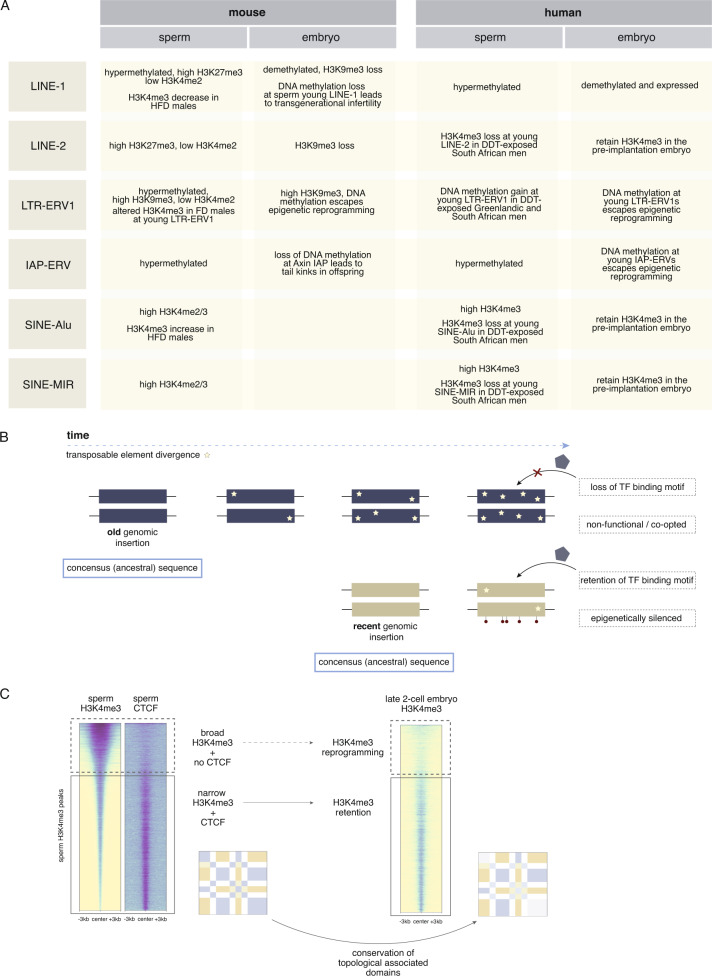
Transcription factors and transposable elements may promote paternal epigenetic inheritance. **A** Table summarizing what is known regarding the enrichment or disruption of epigenetic marks at specific classes of transposable elements in sperm or embryos from mice or men. **B** Diagram illustrating the divergence of transposable element copies after integration in the genome and relationships to transposable element age. For each transposable element integrated in the genome, a percent divergence score can be calculated from its consensus sequence. A low percent divergence score reflects a “young” transposable element that has more recently been integrated in the genome as it has not yet accumulated many sequence substitutions (stars), deletions, insertions, truncations, or undergone recombination. Conversely, a high percent divergence corresponds to an “old” transposable element. These old transposable elements may have diverged too far from the original transcription factor binding site, preventing the recognition by transcription factors and downstream activity. Because young transposable elements retain their recognizable transcription factor binding site, they have higher activity and may be epigenetically silenced if not beneficial to the host. Panel created with BioRender.com. **C** Narrow sperm H3K4me3 peaks that overlap the insulator transcription factor CTCF, important for the 3D chromatin organization in cells, retain H3K4me3 in the pre-implantation embryo. Broad H3K4me3 peaks are not enriched for CTCF in sperm and lose H3K4me3 in the pre-implantation embryo, conferring that CTCF may play a role in the transmission of sperm H3K4me3 post-fertilization. The insulator protein CTCF acts alongside cohesin to regulate topological associated domains in cells by folding domains into loop structures^148^. In line with this, Hi-C experiments have shown a high conservation between the 3D chromatin organization in sperm and in pre-implantation embryos post zygotic genome activation, highlighting the interplay between architectural proteins, and inherited histone modifications in sperm. Panel created with BioRender.com.

The first DNA methylation reprogramming event extends from the zygote to the blastocyst and is characterized by a loss of gamete-specific DNA methylation patterns inherited from the sperm or the oocyte^123^. During this phase, the paternal genome undergoes active demethylation by the TET3 hydroxylase with the accumulation of 5hmC on the paternal genome, whereas the maternal genome is subject to passive demethylation that relies on diluting methylated DNA through cell divisions^124,125^. More recent reports suggest that the maternal genome is also in part actively demethylated during pre-implantation embryogenesis^126^. The DNA methyltransferase DNMT1 was found to be essential for the retention of these methylated regions in the pre-implantation embryo^127^. By the blastocyst stage, DNA methylation reaches its lowest levels but nonetheless, 20% of CpGs still retain gamete-inherited DNA methylation in mice and humans^126,128^. Imprinted regions and evolutionarily young retrotransposons of the IAP and LTR-ERV1 classes resist this DNA methylation reprogramming wave^1,122^ (Fig. 3A, B). Interestingly, about 7% of CpGs in the mouse genome maintain stable methylation states across sperm, the inner cell mass of the blastocyst, and embryonic day E7.5 embryos^126^.

The second mammalian reprogramming event occurs in the primordial germ cells (PGCs) where DNA methylation reprogramming involves a more extensive demethylation event. During this process, a passive DNA methylation phase leads to a global reduction of DNA methylation and is followed by an active demethylation phase by the TET1 and TET2 hydroxylases, whereby DNA methylation from imprinted genes is removed^129^. Imprints are subsequently re-methylated in a gender-specific manner in the gonads by DNMT3A and DNMT3L^75,130^. Between 6–8% of CpGs remain methylated in mice and humans^120,126,131^ and similarly to the pre-implantation embryo DNA demethylation wave, young transposable elements have been shown to resist this second wave of reprogramming (Fig. 3A, B).

### Intracisternal A particles: an example of a transposable element mediating epigenetic inheritance

A landmark example of a paternally-inherited DNA methylation state occurs at an Intracisternal A particle (IAP) and involves the gain-of-function IAP insertion within the sixth intron of the *Axin* gene (*Axin*-fused mice)^132^ (Box 2 and Fig. 3B). *Axin* typically regulates embryo axis formation and in *Axin*-fused mice, the IAP’s DNA methylation levels are directly linked to varying severities of tail kinks. IAP hypomethylation in somatic tissues of *Axin*-fused mice resulted in aberrant expression of *Axin* and a kinky tail phenotype, whereas IAP hypermethylation did not lead to any phenotype. Interestingly, DNA methylation levels in the sperm of *Axin*-fused mice were also hypomethylated and 76% of offspring from males with a kinky tail phenotype also had kinks in their tails. This study implies that sperm DNA methylation levels at the *Axin*-fused locus in part dictate kinky tail phenotype in the progeny^133^, and that the abnormalities transmitted in sperm are propagated and maintained through lineage specification.

More recently, a genome-wide functional dissection of IAPs demonstrated that these transposable elements displayed DNA methylation variability between individual mice of the same genetic background^134^. Although male mice had inter-individual variation in IAP methylation levels in their somatic tissues, these variably-methylated IAPs were fully methylated in their sperm. The hypermethylated state of IAPs in sperm was subsequently remodeled into a variable methylation state in the somatic tissue of their offspring^134^. This suggests that most paternal IAPs undergo methylome reprogramming and indicates that the paternal DNA methylation inheritance of the *Axin*-fused IAP may be an exception rather than the rule. Variably-methylated IAPs were unaltered by maternal exposure to the endocrine disruptor bisphenol A, an obesogenic diet, or methyl donor supplementation^135^. Although paternal effects were not explored in this study, it would be interesting to determine if these variably-methylated IAPs can serve as a mechanism for paternal epigenetic inheritance of environmental effects.

### DNA methylation escapees uncovered by whole-genome bisulfite sequencing

DNA methylation levels between 20–80% have been termed “dynamic CpGs” and exhibit higher sensitivity to environmental exposures, and are predicted to persist in the early embryo (Fig. 2B). These dynamic CpGs are altered in response to folate intake and endocrine disrupting chemicals^114,136^. In fact, men given a high folic acid supplementation showed sperm methylome changes that coincided predominantly to dynamic CpGs^136^. A similar environmental sensitivity was demonstrated in two other populations. In sperm of Greenlandic and South African Indigenous men, high body burdens of *p,p'*-DDE (metabolite of DDT), was associated with dose-like responsive changes in DNA methylation^114^. Over 70% of differentially methylated CpGs occurred at dynamic CpGs and there was a significant overlap between differentially methylated CpGs in both populations. Interestingly, dynamic CpGs that were altered in sperm retained dynamic DNA methylation throughout pre-implantation embryo development (Fig. 2B). These studies confer that dynamic CpGs are more sensitive to environmental insults and may serve as preferential epigenetic transmitter of environmental information escape reprogramming in the earliest stages of development^114^ (Box 3 and Fig. 3A, B).

Even robust proof of transmission of DNA methylation and / or chromatin states from sperm to the embryo is insufficient to provide causal links to alterations in embryonic gene expression at single-locus resolution. Indeed, the sperm methylome and chromatin contexts are highly complex, with multiple epigenetic marks overlapping at widely different functional loci. Little is known regarding how certain marks in sperm may promote splicing variants, alternative promoters, and enhancer-specific transcription, in the embryo. Furthermore, it remains unclear how specific levels of change in DNA methylation and histone modifications alter post-fertilization gene expression. Improving our understanding of how overlapping epigenetic states govern embryo gene expression, will aid in resolving how sperm-inherited epigenetic material influences the embryonic transcriptome. Promising approaches to explore these interrelationships at the mechanistic level include epigenome and DNA editing^137^.

### Evidence for the transmission of sperm non-coding RNAs to embryo

Numerous studies indicate that sperm-derived non-coding RNAs are required for embryo development, can be altered by environmental stressors (Box 3), and are associated with behavioral and metabolic perturbations in the offspring^82,94,96,138–140^. To date, studies have focused on sperm borne non-coding RNAs that originate in the spermatozoa during spermatogenesis^94,99^ and non-coding RNAs acquired during epididymal transit via extracellular vesicle soma-to-germline communications^83,141^. These studies rely on isolating specific populations of non-coding RNAs from the sperm of males exposed to an environmental stressor and injecting them into zygotes to recapitulate the phenotype observed in the offspring of these males^94,96,138,140^. Different classes of small non-coding RNAs in sperm have been suggested to be important for embryo development but how they act as a mechanism of transmission from the sperm to the embryo in terms of modulating transcriptional activation of embryonic gene expression remains largely unknown.

Soma-to-germline communications during epididymal transit allow sperm to assimilate a non-coding RNA payload that is important for embryo development. This transfer of information may occur through extracellular vesicles that are found in the epididymis^141–143^. In one study, sperm transiting from the caput to the cauda epididymis acquired non-coding RNAs that were required for embryo development^142^. Sperm isolated from the caput and injected into the oocyte failed to give rise to viable embryos. Isolating micro-RNA populations from the extracellular vesicles of the cauda epididymis and injecting them into caput-derived zygotes rescued embryo development. However the necessity for sperm to transit through the epididymis to acquire the ability to support embryo development is controversial as sperm retrieved from the testes supports full embryo development. A more recent study failed to replicate the Conine et al. findings and showed that sperm from the caput were fully competent to support embryo development^144^. These discrepancies could be due to differences in mouse strains used or dissection techniques^145^. Suggesting conservation of RNA mediated paternal effects in mammals, a role for sperm-borne RNAs in embryo development has also been demonstrated in cattle; miR-216b levels were lower in sperm from highly fertile bulls and associated with blastocyst development^146^. Importantly, in this study sperm transmission was indicated as miR-216b levels increased in zygotes upon sperm cell entry^146^.

To serve in epigenetic inheritance, sperm non-coding RNAs have to survive passage through the acidic female reproductive tract as well as acrosomal exocytosis which depletes sperm of its small cytoplasmic volume and plasma membrane before fusing with the oocyte. The rapid degradation rate of RNAs deems them unlikely to endure these processes to regulate embryo development. However, evidence suggests that chemical modifications added to non-coding RNAs confer stability. tsRNAs from the sperm of males fed a high-fat diet showed an upregulation in m2G and m5C levels^94^. This later modification is known to ensure tsRNA stability and could therefore mediate the transmission of metabolic phenotypes in the offspring. However, injection of synthetic versions of the most highly expressed tsRNAs in sperm did not produce mice with a metabolic phenotype^94^. In a follow-up study, deletion of the tRNA methyltransferase DNMT2 prevented an increase of m2G and m5C on the sperm tsRNAs from males fed a high-fat diet^82^. This consequently abolished the transmission of a paternal high-fat diet induced metabolic disorders to the offspring via tsRNAs^82^.

Most studies linking non-coding RNAs to phenotypes in offspring have relied on sperm-purified or synthetic RNA injected into the zygote^94,96,138,140^. To unequivocally demonstrate transmission, non-coding RNAs should be tagged in sperm and tracked at fertilization to show that they persist in the early embryo. In addition, it is not clear how non-coding RNAs influences gene expression to alter offspring phenotypes. A thorough dissection of the regulatory origins of non-coding RNAs’ must be addressed to understand whether non-coding RNA differences in sperm due to an environmental stress, are a consequence of an upstream epigenetic mechanism such as DNA methylation or histone modifications.

## Genomic factors that influence epigenetic inheritance

Why certain regions that bear DNA methylation or histone modifications in sperm are retained in the embryo and others are reprogrammed is unknown. The mechanisms enabling a post-fertilization retention of epigenetic memory may be influenced by the underlying DNA sequence itself (Fig. 3). Distinct classes of young transposable elements have been shown to preferentially associate with the inheritance of epigenetic material from sperm (Fig. 3A, B). Furthermore, specific motifs, transcription factors or chromatin modifiers have been associated with the retention of sperm epigenetic factors in the embryo (Fig. 3C).

### Sperm harbors multiple classes of transcription factors

Recent studies suggest that sperm transmit a transcriptional map at fertilization that may cooperate with histone methylation in embryonic gene regulation. A number of key transcription factors are enriched in sperm at developmental regions and co-localize to various epigenetic marks involved in epigenetic inheritance. For example, CTCF and the cohesin subunit SMC1 are enriched in sperm at regions that bear H3K4me1 and H3K4me3, suggesting a coordinated involvement in long-range interactions to organize the three-dimensional architecture in sperm^9,45,147^. Remarkably, ~23,000 CTCF sites in sperm are also present in ESCs^45^. These transcription factors work together to organize the three-dimensional structure of the genome^148^. Regions marked by H3K4me3, CTCF, and SMC1 in sperm, retain H3K4me3 in the 2-cell embryo at the same genomic loci. Conversely, regions marked by H3K4me3 in sperm but devoid of CTCF and SMC1, are not enriched for H3K4me3 in the 2-cell embryo, suggesting that these transcription factors may be insulating sperm H3K4me3 from post-fertilization reprogramming^9^ (Fig. 3c).

In support of this possibility, in mouse testicular germ cells, the ATF7 transcription factor binds to over 3,000 sites and recruits the H3K9me2 and H3K9me3 methyltransferases G9A, SUV39H1, and ESET/SETDB1^149^. A paternal low protein diet causes ATF7 activation and H3K9me2 reduction in germ cells, leading to deregulated gene expression in offspring livers. Interestingly, offspring of males with a mutated *Atf7* that were fed a low protein diet did not exhibit changes in their liver gene expression. This study suggests that ATF7 is required for hepatic gene expression changes induced by a paternal low-protein diet^149^.

### Transposable element regulation in paternal epigenetic inheritance

The genome is comprised of almost 50% repetitive elements and it has recently come to light that their expression is tightly regulated by both DNA methylation and chromatin content in early embryonic development^150^ (Box 2 and Fig. 3A, B). During pre-implantation development and PGC migration, LINE-1 elements are substantially demethylated whereas IAP elements are largely resistant to reprogramming^151^. Indeed, the highly active and thus hazardous IAP subclass IAPLTR1, stays significantly more methylated than other loci during PGC development^152^ (Fig. 3A). The specific machinery used to maintain 5mC at IAPLTR1s and ensure robust genomic integrity during PGC reprogramming is unknown. In pre-implantation embryos, precocious silencing of LINE-1 activation leads to developmental delay^153^. Remarkably, the origins of its embryonic regulation has been linked to the sperm epigenome. Broad H3K9me3 domains, a mark that typically suppresses transposable elements, are enriched in sperm heterochromatin^46^, and are predominately lost from the paternal allele of the pre-implantation embryo coinciding with the expression of LINE elements^109^ (Fig. 3A). Intriguingly, H3K4me3 also co-localizes to transposable elements, suggesting that this active mark may contribute to transposable element gene activation during embryogenesis. In support of the paternal epigenome in transposable element regulation is that abnormal PRC2 function during germ cell development leads to altered H3K27me3 in male gametes at repetitive sequences and a subsequent deregulation of retrotransposed pseudogenes in eight-cell embryos^61^ (Fig. 3A).

DNA methylated regions that escape reprogramming in the embryo co-localize to young transposable elements suggesting they may be preferential genomic elements for the transmission of environmentally altered epigenetic information across generations^154^. The age of a transposable element can be extrapolated from the amount of time the transposable element has been integrated into genome (Fig. 3B). An “old” transposable element will accumulate mutations and have a high percent divergence score relative to the ancestral transposable element sequence. Conversely, a low percent divergence score characterizes a “young” transposable element which will be more active in the genome. Transposable elements influence gene expression via different mechanisms notably by behaving as promoters or enhancers and serving as transcription factor binding sites, modifying the 3D chromatin architecture, producing a range of non-coding RNAs, encoding proteins that facilitate the emergence of new gene regulatory networks, and promoting the heritable and long-term silencing of surrounding DNA^150^. For example, there is an over-representation of SINEs at developmental promoters marked by H3K4me3 in human sperm^44,114^.

Adding further support to the importance of young transposable elements in epigenetic inheritance comes from an MTHFR-deficient mouse model; loss of sperm DNA methylation at young LINE-1 transposable elements coincided with a decline in fertility across two generations, suggesting that the re-activation of these transposable elements impaired fertility intergenerationally^155^ (Fig. 3A). In sperm from DDT-exposed Greenlandic and South African men, regions with altered DNA methylation and H3K4me3 enrichments were preferentially found at young transposable elements including, LTR-ERV1 (differentially methylated regions) and LINE-2, SINE-MIR, SINE-Alu (differentially enriched H3K4me3 peaks)^114^. Notably these alterations were predicted to be retained in the pre-implantation embryo and occurred at transposable elements associated with genes enriched for neurodevelopment. Implicating these changes in epigenetic transmission of disease is the increased incidence of autism and learning disorders in DDT-exposed populations^114^ (Fig. 3A,B).

These findings raise an intriguing question: why might epigenetic modifications at young transposable elements be preferential carriers of epigenetic information across the paternal germline? Repressive marks such as DNA methylation are known to be critical for silencing the parasitic activity of newly integrated transposable elements^119,122^. Derepression of these transposable elements has been implicated in disease such as cancer, schizophrenia, and other complex diseases^24,156–158^. Because of their young evolutionary age, it is possible that these transposable elements have not yet been co-opted by the host and are thus deemed non-beneficial. In this case, young transposable elements may retain their DNA methylation levels during early embryo development. As environmental cues impact the sperm methylome, DNA methylation alterations at loci that resist reprogramming could impact embryonic gene expression. Other studies postulate that because young transposable elements have not yet diverged from their ancestral sequence, their intact transcription factor binding sites can still be co-regulated by multiple transcription factors such as CTCF, NANOG, and OCT4^159,160^. As we have previously demonstrated, regions with narrow H3K4me3 in sperm that retain H3K4me3 in the 2-cell embryo are bound by CTCF in sperm^9^ (Fig. 3C). It is therefore possible that these transcription factors are required in early development to insulate certain transposable elements from epigenetic reprogramming and to poise them for the earliest stages of embryo development.

## Influence of sperm-inherited epigenetic factors on embryonic gene expression

**Fig. 4:**
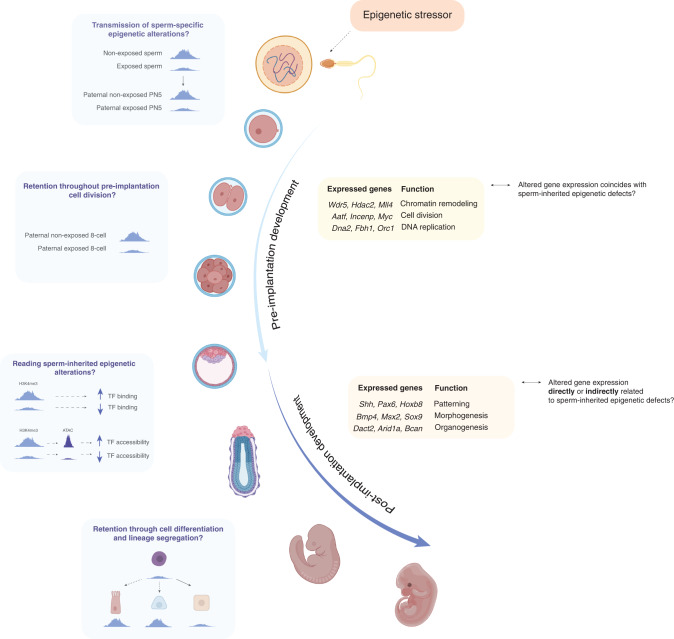
Moving from association to function in the field of paternal epigenetic inheritance. Overview of remaining questions that will permit the field of paternal epigenetic inheritance to understand how transmitted epigenetic marks elicit changes in embryo development intergenerationally and transgenerationally. While the molecular mechanisms underlying epigenetic inheritance are partially resolved there remain unanswered questions. Current studies suggest that exposures can alter epigenetic marks including DNA and histone methylation (H3K4me3), some of which may alter transcription factor binding and consequently chromatin organization. These epigenetic changes are partially retained on the paternal chromatin where they alter transcription in the early embryo leading to downstream phenotypes. It is unclear what marks may persist through lineage segregation and cell differentiation. Boxed are examples of genes that have altered H3K4me3 and / or DNA methylation in folate deficient mouse sperm^9^ or DDT-exposed South African human sperm^114^. As highlighted, genes with altered epigenetic marks are involved in both pre- and post-implantation development. Whether genes that are expressed at later stages of development carry sperm-inherited epigenomic alterations throughout embryogenesis until their expression, has never been explored. How sperm RNA content is altered in relation to chromatin and DNA methylation is unknown as is how sperm-transmitted RNA leads to altered embryonic gene expression. Figure created with BioRender.com.

How do alterations in sperm chromatin or DNA methylation lead to changes in gene expression in the early embryo, and are they retained throughout embryogenesis to be aberrantly expressed at later stages? (Fig. 4). For changes in the sperm epigenome to elicit consequences on the next generation, they must be transmitted, and escape reprogramming post-fertilization and elicit changes in gene expression to alter developmental pathways in the embryo. In this section we discuss how inherited sperm epigenetic marks could regulate embryonic gene expression to alter development.

### Is the sperm transcriptional machinery transmitted to the embryo?

Although transcriptionally inert, mature sperm bears numerous classes of transcription factors that may reflect residual binding from spermatogenesis or be later solicited during embryogenesis if transmitted^45,147^. In sperm, two phosphorylated forms of RNA polymerase II (RNAPIISer5ph and RNAPIISer2ph) are enriched at transcriptional start sites and at putative enhancers marked by H3K4me1 and H3K27ac. Mediator, a stabilizer of the transcription complex, also co-localizes to certain promoters with RNAPII. Genes bearing transcription complexes in sperm are not transcribed in round spermatids, entailing that they may poise gene expression post-fertilization, or be a memory of prior expression in spermatogenesis. Interestingly, active loci in sperm are typically bound by transcription factors in the pre-implantation embryo^147^. The pioneer transcription factor FOXA1 (facilitates the binding of estrogen and androgen hormone receptors) is also enriched at sperm transcriptional start sites and in intergenic space^147^. In  ESCs, FOXA1 was required to activate transposable elements to promote differentiation^161^. Understanding the binding affinity of these transcription factors to chromatin and determining whether they are transmitted post-fertilization, will be important to uncover whether their presence in sperm prime early embryo gene expression.

### Setting up the embryo chromatin architecture

Hi-C experiments have demonstrated that sperm chromatin is organized into distinct topological associated domains^45,162,163^ (Fig. 3C). This specific arrangement includes a high number of extra-long-range interactions (over 2 million base pairs apart), whereby more than 50% of interactions in sperm occur between different TADs^163^. The higher-order organization in sperm differs from the lack of TADs in mature MII oocytes. Although TAD structures in zygotes and two-cell embryos are obscure, chromatin organization becomes re-established during subsequent stages of pre-implantation embryo development^163^. Certain studies highlight the resemblance between sperm domains and mouse ESC or late-2-cell embryo chromatin architectures^45^. Single-cell Hi-C experiments in hybrid embryos show that a subset of TADs on the paternal allele of the 1-cell and 2-cell embryo are paternally-biased and may be inherited from sperm^164^. Interestingly, CTCF a master regulator of chromatin looping and domain establishment, is highly enriched at domain boundaries in both sperm and mouse ESCs. The boundary-specific CTCF enrichment co-occurs with the active marks H3K4me3, H3K9ac, and H3K27ac^9,45^. Narrow H3K4me3 peaks in sperm marked by CTCF retain H3K4me3 in the 2-cell embryo. These finding are consistent with the role of CTCF to prevent H3K4me3 spreading in somatic cells^165^ and may permit the narrow sperm H3K4me3 peaks to position themselves into the concordant H3K4me3 domain dips of the oocyte, similarly to a key and lock mechanism (Figs. 2A and 3A). Further describing the interactions between histone modifications and domain formations in sperm, and whether they mediate embryonic chromatin architecture to regulate gene expression, will provide a robust functional link between sperm epigenetic inheritance and embryo gene expression.

### Interactions between sperm and egg cellular contents post-fertilization

Exploring how sperm or oocyte proteins influence the maternal or paternal alleles respectively, improves our understanding of how reprogramming events are initiated in the early embryo. To date this information is limited to a few studies that have examined this phenomenon of allelic influence. For example, several dozen CpG-rich promoters are de novo methylated by maternal DNMT3A on the paternal allele of the 2-cell embryo. Loss of maternal DNMT3A leads to a premature transcription of these paternally-expressed genes at the 4-cell stage^166^. In another study, the oocytes of hyperglycemic mice or from humans with diabetes, showed reduced expression of *Tet3* deoxygenase. This was associated with the hypermethylation of paternal alleles at multiple insulin secretion genes and glucose intolerance in the progeny^167^. Finally, it has been shown that the oocyte-provided SRPK1 phosphorylates protamines in sperm to initiate protamine-to-histone exchange and paternal genome reprogramming. Lack of maternal SRPK1-mediated protamine removal leads to an inefficient HIRA recruitment and H3.3 deposition on the paternal pronucleus^52^.

### Are epigenetic alterations in sperm maintained throughout development?

Embryogenesis is a highly intricate process that involves cell divisions, cell differentiations, lineage specifications, and vast epigenetic remodeling events, with stage and spatial specificities. In genetic or environmental models of epigenetic inheritance, many genes carrying altered histone or DNA methylation in sperm, are not turned on in the pre-implantation embryo but become expressed during later stages of embryogenesis such as gastrulation and organogenesis^8,9,41,43^. Whether sperm-inherited epigenetic defects at developmental regulatory regions can be retained throughout embryogenesis until they become activated is unknown. We propose two models that may explain the development of complex diseases and birth defects from an aberrant sperm epigenome. Firstly, paternally inherited phenotypes may result from a cascade effect of genes that were deregulated in the pre-implantation embryo due to abnormal sperm-inherited epigenetic marks. Secondly, and not mutually exclusive from our first model, certain genes may retain their aberrations throughout cell divisions and differentiations, and only provoke alterations in gene expression at later stages of embryogenesis when they typically become expressed (Fig. 4).

### Cross-talk between epigenetic mechanisms and relay of information during development

Certain studies suggest that an information transfer occurs between histone modifications and DNA methylation in the embryo. Noncanonical oocyte-derived H3K27me3 imprinting was found to be transient in the pre-implantation embryo and necessary for DNA methylation establishment and subsequent maintenance in extraembryonic lineages. Interestingly, the de novo DNA methylation was associated with paternal allele H3K4me3 establishment in the pre-implantation embryo^168^. In a paternal epigenetic inheritance study, H3K27me3 changes in sperm were associated with DNA methylation changes in the tissue of the developed offspring, suggesting a potential relay of epigenetic information between sperm histones and DNA methylation after the DNA methylation reprogramming wave during pre-implantation development^62^. Interestingly, sperm-specific H3K27me3 is lost on the paternal allele of the pre-implantation embryo therefore it is unclear how paternally-inherited bivalency is conserved throughout early embryogenesis. It is possible that other more stable epigenetic marks such as DNA methylation relay this silencing.

## Concluding remarks

The field of paternal epigenetic inheritance has remained stalled on association-based studies that drew parallels between environmentally-induced alterations in the sperm and a phenotype in the offspring. Although valuable in pushing our understanding on how environmental stressors impact specific loci in the sperm epigenome, these studies have not resolved whether these marks contributed to development in the next generation. There exists compelling evidence conferring a role for certain transcription factors and classes of transposable elements in regulating epigenetic inheritance through the paternal germline. Understanding how these genomic factors interact with the sperm epigenome will untangle how certain marks in sperm escape reprogramming. Furthermore, most paternal epigenetic inheritance studies to date have focused on a single epigenetic factor acting unilaterally. It is clear that epigenetic marks in sperm overlap and may act in concert during embryogenesis. Although a growing number of studies have identified mechanisms mediating intergenerational epigenetic inheritance from sperm to the embryo, evidence for epigenetic factors that propagate environmentally-induced phenotypes transgenerationally through the paternal mammalian germline is still lacking. As studies in mouse models are beginning to determine which marks in sperm transmit epigenetic information to the offspring, and how these inherited marks deregulate embryonic gene expression and subsequent development, we can apply this knowledge to human studies to identify sensitive regions of the sperm epigenome and decipher whether they are at the root of complex diseases and developmental disorders.

## Data Availability

Mouse sperm ChIP-seq datasets from Figs. 1B, 2A, and 3C were retrieved from (GEO: GSE135678 for H3K4me3^9^, GEO: GSE79230 for H3K4me1 and H3K27ac^45^, GEO: GSE145679 for H3K27me3^54^, GEO: GSE55471 for H3K4me2^21^, GEO: GSE153531 for H2AK119ub1^48^, GEO: GSE112835 for H3K36me3^63^; GEO: GSE97778 for H3K9me3^46^). Mouse sperm PANDORA-seq datasets from Fig. 1B were retrieved from (GEO: GSE144666^99^). Mouse sperm CTCF ChIP-seq dataset from Fig. 3C was retrieved from (GEO: GSE79230^45^). Oocyte and two-cell pre-implantation embryo chromatin ChIP-seq dataset from Figs. 2A and 3C were retrieved from (GEO: GSE73952 for H3K4me3 and H3K27me3^110^, GEO: GSE72784 for H3K27ac^111^, GEO: GSE112835 for H3K36me3^63^; GEO: GSE97778 for H3K9me3^46^). Human Greenlandic and South African sperm Methyl-Capture-seq^114^ (DNA methylation) from Fig. 2B will be made available upon request via material transfer agreement only.
